# Tracking Pedestrians across Multiple Microcells Based on Successive Bayesian Estimations

**DOI:** 10.1155/2014/719029

**Published:** 2014-08-11

**Authors:** Yoshiaki Taniguchi, Masahiro Sasabe, Takafumi Watanabe, Hirotaka Nakano

**Affiliations:** ^1^Faculty of Science and Engineering, Kindai University, Higashiosaka 577-8502, Japan; ^2^Nara Institute of Science and Technology, Ikoma 630-0192, Japan; ^3^Graduate School of Information Science and Technology, Osaka University, Suita 565-0871, Japan; ^4^Cybermedia Center, Osaka University, Toyonaka 560-0043, Japan

## Abstract

We propose a method for tracking multiple pedestrians using a binary sensor network. In our proposed method, sensor nodes are composed of pairs of binary sensors and placed at specific points, referred to as gates, where pedestrians temporarily change their movement characteristics, such as doors, stairs, and elevators, to detect pedestrian arrival and departure events. Tracking pedestrians in each subregion divided by gates, referred to as microcells, is conducted by matching the pedestrian gate arrival and gate departure events using a Bayesian estimation-based method. To improve accuracy of pedestrian tracking, estimated pedestrian velocity and its reliability in a microcell are used for trajectory estimation in the succeeding microcell. Through simulation experiments, we show that the accuracy of pedestrian tracking using our proposed method is improved by up to 35% compared to the conventional method.

## 1. Introduction

In recent years, many researchers and developers have focused on sensor networks that consist of lots of sensors with wireless communication devices. Among sensor network applications, pedestrian tracking is one of the most promising applications. Pedestrian tracking technologies are significant for realizing safe and secure societies: preventing accidents in health care facilities; detecting strangers in public or private spaces. They also enable us to analyze human behavior in event areas or commercial establishments.

Binary sensors are among the simplest and inexpensive sensors, and they can only detect the presence or absence of pedestrians in its sensing region. By deploying multiple binary sensors, information on the numbers of pedestrians or the trajectories of pedestrians can be estimated. There are several studies on pedestrian tracking using binary sensor networks [1–6]. However, in these studies, they assume that a pedestrian does not change its velocity in the monitoring area. In addition, they also assume that sensor nodes are distributed uniformly so that the sensing region covers the entire monitoring area. In actual monitoring areas, there are multiple points where pedestrians temporarily change their movement characteristics, such as doors, stairs, and elevators. We call these points gates. Furthermore, most studies on pedestrian tracking using binary sensor networks aim at single-pedestrian tracking.

**Figure 1:**
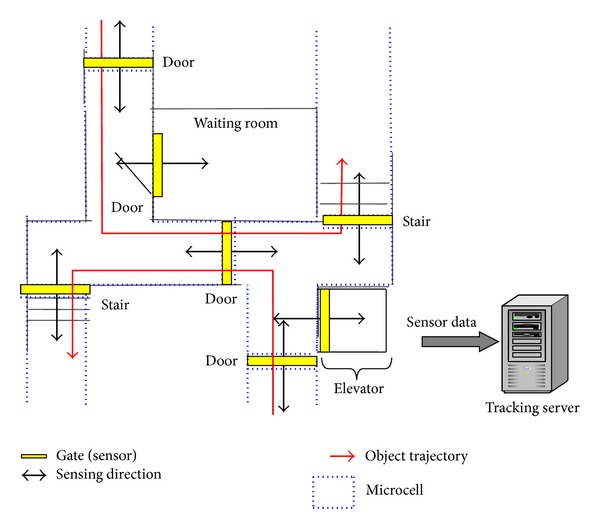
Overview of the pedestrian tracking system.

In this paper, we consider a pedestrian tracking system where sensor nodes are placed only at gates so that the monitoring region is divided into multiple smaller regions referred to as microcells, as shown in Figure 1. We assume that each gate can detect pedestrian arrival or departure events with the pedestrians' moving directions using a pair of binary sensors [7–11]. Our system focuses on pedestrian tracking in a building, where multiple pedestrians move. Sensor information is collected to a tracking server through wireless networks and the tracking server estimates pedestrian trajectories.

In [12], we proposed a Bayesian estimation-based pedestrian tracking method in microcells based on investigation of actual pedestrian trajectories in a microcell. The method focuses on pedestrian tracking in a single microcell. In the method, pedestrian tracking is conducted by matching the pedestrian gate arrival and gate departure events using statistically obtained information on pedestrian velocities. In [12], the effectiveness of the Bayesian estimation-based method was shown by comparative evaluation with a combinatorial optimization-based method.

In this paper, we propose a method for tracking pedestrians across multiple microcells. In our proposed method, pedestrian tracking in each microcell is conducted based on the conventional Bayesian estimation-based method [12] with extension. To improve accuracy of pedestrian tracking, the tracking server records velocity information, which consists of estimated pedestrian velocity and its reliability, in a microcell. The tracking server uses velocity information for estimating the trajectory of the pedestrian in the succeeding microcell. We evaluate the performance of our proposed method through simulation experiments.

The rest of this paper is organized as follows. In Section 2, we introduce related work. In Section 3, we propose a method for tracking pedestrians across multiple microcells. We evaluate the performance of our proposed method through simulation experiments in Section 4. Finally, we conclude this paper with outlook on future research in Section 5.

## 2. Related Work

Tracking multiple pedestrians has received a great attention in the fields of computer vision and sensor networks. There are several studies on tracking multiple pedestrians, such as Active Badge [13], Active Badge Location System [14], and Cricket [15]. In Active Badge Location System [14], multiple receivers receive the signal sent from the wireless device equipped with a pedestrian. The information is collected to a server, and it estimates the location of the pedestrian based on the differences among the received times. This system requires a unique identifier for each pedestrian that results in high deployment costs.

Multiple hypothesis tracker (MHT) [16] can achieve pedestrian tracking without unique identifiers. It first calculates all possible states based on observation results at sensors. Here, the state is represented as the number of pedestrians and their trajectories in the monitoring area. Then, it estimates the current state based on the occurrence probability of each state that is calculated in advance. However, it is a NP hard problem because state explosion occurs with increase of pedestrians. Markov chain Monte Carlo (MCMC) method [17] can cope with this problem. However, the method needs high-end sensors that can detect the number of pedestrians in their sensing regions while distinguishing the color, shape, and velocity of each pedestrian.

Pedestrian tracking using inexpensive binary sensors has been also attracting many researchers. However, most of the work aims at single-pedestrian tracking. In [1], the authors proposed a tracking method for multiple pedestrians based on Particle Filer Algorithm [18, 19]. Particle Filer Algorithm first predicts all possible next states from the current state. The state is the same as that in MHT. Then, it calculates the likelihood for each estimated next state using the observation results from the sensors. It selects a few states in a descending order of the likelihood from the states. These procedures are continued to track pedestrians successively. However, this method assumes that a pedestrian does not change its velocity in the entire monitoring area.

In this paper, we also propose a method for tracking multiple pedestrians in a monitoring area using a binary sensor network, especially focusing on pedestrian tracking in a building. As mentioned before, we divide the monitoring area into multiple microcells by placing sensor nodes at specific points, that is, gates. By sharing pedestrian velocity information among microcells, we try to improve the accuracy of pedestrian tracking.

## 3. Proposed Method for Tracking Pedestrians across Multiple Microcells

In this section, we propose the pedestrian tracking method.

### 3.1. Basic Behavior

Figure 1 shows the overview of the tracking system. We suppose that there are lots of pedestrians in the monitoring area and the number of transit microcells for a pedestrian is limited. In actual monitoring area, there are multiple points where pedestrians temporarily change their movement characteristics, such as doors, stairs, and elevators. In this paper, we call these points gates. In the tracking system, sensor nodes are placed at gates so that the monitoring region is divided into multiple smaller regions referred to as microcells. A sensor node is composed of a pair of binary sensors with a wireless communication device, and it detects pedestrian arrival events and departure events. We denote an arrival event observed at sensor node on gate *g*_*a*_ in microcell *m*_*x*_ at time *t*_*i*_ as *e*_arr_^(*x*)^(*g*_*a*_, *t*_*i*_) and a departure event observed at sensor node on gate *g*_*d*_ in microcell *m*_*y*_ at time *t*_*j*_ as *e*_dep_^(*y*)^(*g*_*d*_, *t*_*j*_). For each arrival event in intermediate microcells, velocity information is maintained as explained later. We should note here that a departure event in an intermediate microcell corresponds to another arrival event, referred to as corresponding arrival event, in the succeeding microcell. Sensor information on arrival/departure event and time of event is collected in the tracking server through wireless networks, and the tracking server estimates pedestrian trajectories based on sensor information.

When the tracking server obtains information on arrival event in microcell *m*_*x*_, it adds the arrival event to the set of candidate arrival events *E*_arr_^(*x*)^ for future matching. On the other hand, when the tracking server obtains information on departure event in microcell *m*_*x*_, it estimates trajectory of pedestrian by matching the departure event and arrival events in the set of candidate arrival events *E*_arr_^(*x*)^ using a Bayesian estimation-based method. The matching method is based on the conventional method with modification to handle velocity information. The details are explained in Section 3.2. After the estimation, the tracking server records the velocity information to the corresponding arrival event for future estimations of the pedestrian in the succeeding microcell. Here, the velocity information is composed of mean velocity $\bar{v}$ and deviation of velocity *σ*. The details for obtaining velocity information are explained in Section 3.3.

**Figure 2:**
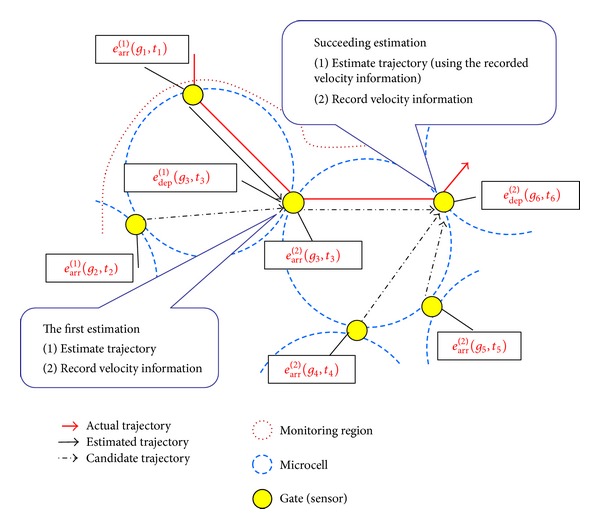
Example of behavior of our proposed method.

Figure 2 illustrates an example of behavior of our proposed method. In Figure 2, when the tracking server obtains information on departure event *e*_dep_^(1)^(*g*_3_, *t*_3_) in microcell *m*_1_, it estimates the pedestrian trajectory by matching the departure event and arrival events in the set of candidate arrival events *E*_arr_^(1)^ = {*e*_arr_^(1)^(*g*_1_, *t*_1_), *e*_arr_^(1)^(*g*_2_, *t*_2_)} in microcell *m*_1_. In this example, trajectory from gate *g*_1_ to gate *g*_3_ is estimated. After the estimation, it records velocity information to the corresponding arrival event *e*_arr_^(2)^(*g*_3_, *t*_3_) in the succeeding microcell *m*_2_. When the tracking server obtains information on departure event *e*_dep_^(2)^(*g*_6_, *t*_6_) in microcell *m*_2_, it estimates the pedestrian trajectory in microcell *m*_2_ using the recorded velocity information if it is available. In the following sections, we explain the details of our proposed method.

### 3.2. Bayesian Estimation-Based Pedestrian Tracking

When the tracking server obtains information on departure event *e*_dep_^(*x*)^(*g*_*d*_, *t*_*j*_), it starts for matching between the departure event and arrival events in the set of candidate arrival events *E*_arr_^(*x*)^ based on the distribution of pedestrian velocities $N(\bar{v},\sigma^{2})$ and probabilities of gate transitions between two gates. More precisely, the tracking server calculates the matching likelihood *p*(*e*_arr_^(*x*)^(*g*_*a*_, *t*_*i*_)∣*e*_dep_^(*x*)^(*g*_*d*_, *t*_*j*_)) for arrival event *e*_arr_^(*x*)^(*g*_*a*_, *t*_*i*_) ∈ *E*_arr_^(*x*)^ in microcell *m*_*x*_. The matching likelihood is the probability that the departure event *e*_dep_^(*x*)^(*g*_*d*_, *t*_*j*_) corresponds to an arrival event *e*_arr_^(*x*)^(*g*_*a*_, *t*_*i*_) and is calculated based on the Bayes theorem as follows [12]:

(1) p(earr(x)(ga,ti) ∣ edep(x)(gd,tj)) =ptrn(x)(ga,gd)ptm(x)(tj−ti,d(x)(ga,gd)),

where *p*_trn_^(*x*)^(*g*_*a*_, *g*_*d*_) is the gate-transition probability that a pedestrian arrives at gate *g*_*a*_ and departs from gate *g*_*d*_ in microcell *m*_*x*_. *d* ^(*x*)^(*g*_*a*_, *g*_*d*_) is the distance between gate *g*_*a*_ and gate *g*_*d*_ in microcell *m*_*x*_. *p*_tm_^(*x*)^(*τ*, *d*) is probability density function of the pedestrian transit time required for a pedestrian to cover a distance *d* and is as follows:

(2) $$\begin{array}{c} p_{\text{tm}}^{\left(x\right)}\left(\tau ,d\right)=\frac{d}{\sqrt{2\pi }\sigma \tau^{2}}\exp\left(-\frac{{\left((d/\tau )-\bar{v}\right)}^{2}}{2\sigma^{2}}\right). \end{array}$$

Velocity information, that is, mean velocity $\bar{v}$ and deviation of velocity *σ*, is recorded for each arrival event at the timing of previous estimation. We explain the details in the next section. For arrival events in edge microcells, default values ${\bar{v}}_{0}$ and *σ*_0_ are used for mean velocity $\bar{v}$ and deviation of velocity *σ*, respectively. Parameteres ${\bar{v}}_{0}$ and *σ*_0_ are assumed to be obtained preliminarily. In addition, the distribution of gate-transition probabilities *p*_trn_^(*x*)^(*g*_*a*_, *g*_*d*_) and the distribution of gate distances *d* ^(*x*)^(*g*_*a*_, *g*_*d*_) are assumed to be obtained preliminarily.

The tracking server selects one arrival event that has the maximum value of matching likelihood for the pedestrian trajectory.

### 3.3. Obtaining Velocity Information for Successive Estimations

After matching, the tracking server records velocity information, that is, mean velocity $\overset{-}{v}$ and deviation of velocity *σ*, as follows. Suppose that the tracking server selects the arrival event *e*_arr_^(*x*)^(*g*_*c*_, *t*_*k*_) as the estimation result for the departure event *e*_dep_^(*x*)^(*g*_*d*_, *t*_*j*_) in microcell *m*_*x*_. We first define the reliability of the estimation result, referred to as matching reliability *r*, as follows:

(3) $$\begin{array}{c} r=\frac{p\left(e_{\text{arr}}^{\left(x\right)}\left(g_{c},t_{k}\right)\;|\;e_{\text{dep}}^{\left(x\right)}\left(g_{d},t_{j}\right)\right)}{\sum_{e_{\text{arr}}^{\left(x\right)}\left(g,t\right)\in E_{\text{arr}}^{\left(x,\alpha \right)}}^{}p\left(e_{\text{arr}}^{\left(x\right)}\left(g,t\right)\;|\;e_{\text{dep}}^{\left(x\right)}\left(g_{d},t_{j}\right)\right)}. \end{array}$$

Here, *E*_arr_^(*x*,*α*)^ is a set of the top *α*  (1 ≤ *α*) arrival events in the order of matching likelihood. Matching reliability *r* ranges [1/|*α*|, 1]. The estimation result is more reliable when *r* is high.

The deviation of velocity *σ* is calculated based on the estimation reliability *r* as follows:

(4) $$\begin{array}{c} \sigma =\left(1-r^{\beta }\right)\sigma_{0}. \end{array}$$

Here, *β*  (1 ≤ *β*) is a parameter to control the randomness of velocity. Small values of *β* enlarge the randomness. In (4), the deviation of velocity *σ* exponentially decreases with the increase of the matching reliability *r*. This characteristic indicates that the estimation accuracy of pedestrian tracking in the succeeding microcell steeply improves when pedestrian tracking in the previous microcell is successesful.

On the other hand, the mean velocity $\overset{-}{v}$ is calculated as follows:

(5) $$\begin{array}{c} \bar{v}=\frac{d^{\left(x\right)}\left(g_{c},g_{d}\right)}{t_{i}-t_{k}}. \end{array}$$

## 4. Simulation Experiments

In this section, we evaluate our proposed method through simulation experiments. In this paper, to evaluate fundamental performance of our proposed method, we use an artificial dataset as explained in the following.

### 4.1. Simulation Settings

First, we explain the microcell model and the pedestrian mobility model. In our system, the monitoring area is divided into multiple microcells. At a steady state of the system, we can expect that the accuracy of pedestrian tracking in a microcell is almost the same as that in the entire monitoring area. Thus, we focus on one microcell in this paper and we use following models.

The distance and transition probability between two arbitrary gates in a microcell in actual environment are nonuniform as reported in [12]. In this paper, they are determined randomly as shown in Tables 1 and 2 to represent the nonuniform characteristics. We set the number of gates in the microcell to five. Here, we note that the evaluation results are affected depending on the constitution of microcell. Detailed evaluation by changing the parameters of microcell is one of our future works.

We assume that new pedestrians arrive to the monitoring area following a Poisson distribution with the mean arrival rate of *λ* = 0.16 [pedestrians/s]. This is because the distribution of pedestrian arrival is often assumed as a Poisson distribution [9, 12, 20–22]. The pedestrian's arrival gate is selected among five gates uniformly. The pedestrian's departure gate is determined according to transition probabilities in Table 2. When a pedestrian departs from the microcell, it again enters to the microcell whose gate is selected among five gates uniformly. A pedestrian departs from the monitoring area when the pedestrian transits through a fixed number of microcells *n*. The velocity of pedestrian follows a normal distribution $N\left({\overset{-}{v}}_{0},\sigma_{0}^{2}\right)$ where ${\overset{-}{v}}_{0}$ and *σ*_0_ are set to 1.31 [m/s] and 0.272, respectively.

To evaluate the effects for determining the deviation of velocity based on the matching reliability, we also conduct simulations using the following methods.

(i) Comparative method: in the comparative method, instead of (4), the deviation of velocity *σ* is calculated as follows:
  (6) $$\begin{array}{c} \sigma =\gamma \sigma_{0}. \end{array}$$
 Here, *γ*  (0 ≤ *γ* ≤ 1) is a parameter to control the randomness of velocity. Small values of *γ* decrease the randomness. For the mean velocity $\overset{-}{v}$, the same equation, that is, (5), is used in the comparative method.
(ii) Conventional method [12]: in the conventional method, pedestrian tracking in microcells is conducted independently without obtaining velocity information. More precisely, default values ${\overset{-}{v}}_{0}$ and *σ*_0_ are always used for mean velocity $\overset{-}{v}$ and deviation of velocity *σ*, respectively, in the conventional method.

As an evaluation index, we define tracking success ratio as the ratio of the number of successful estimations to the total number of estimations. To evaluate our proposed method in a steady state, we conduct a 4000 [s] simulation and use the average in the last 2000 [s] in the following evaluations.

### 4.2. Evaluation on the Optimum Parameter Settings

**Figure 3:**
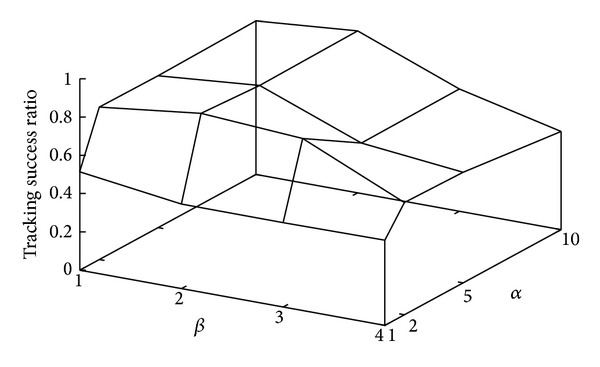
Relationship between parameter settings and tracking success ratio of our proposed method.

We first investigate the optimum parameter settings of our proposed method. The number of transit microcells *n* is set to 11.  Figure 3 depicts the relationship between parameters *α*, *β* and tracking success ratio of our proposed method. As shown in Figure 3, the tracking success ratio is lower independently of *β* in case of *α* = 1. This is because the matching reliability cannot be accurately calculated using (3). In this case, the matching reliability is always one for any estimation result. On the other hand, the results almost do not change in case of *α* ≥ 2. This is because the matching likelihood of arrival events in (3) is almost zero except for the first and second arrival events in the simulations. We can conclude that *α* = 2 is sufficient to achieve a high tracking success ratio while suppressing the processing overheads for the estimation.

It is also shown that *β* should be set to two since the tracking success ratio is the maximum as shown in Figure 3. The larger *β*, the larger the randomness added to the estimated velocity regardless of the estimation reliability. As a result, the tracking success ratio decreases when *β* is large. In the following evaluations, we use *α* = 2 and *β* = 2.

### 4.3. Effect of the Distribution of the Number of Transit Microcells

We next investigate the effect of distribution of the number of transit microcells. We also confirm how the simulation reaches a steady state in this section.

In the previous section, we used a uniform distribution for the number of pedestrians' transit microcells. In actual monitoring areas, the number of transit microcells is different for each pedestrian. There may be a situation where most of pedestrians transit only a few microcells due to the characteristics of the building. To evaluate the effect of the distribution of the number of transit microcells *n*, we conduct simulations where the number of transit microcells *n* follows a nonuniform distribution. In this paper, as a nonuniform distribution, we use a Zipf distribution since it deals with a strong bias of distribution.

**Figure 4:**
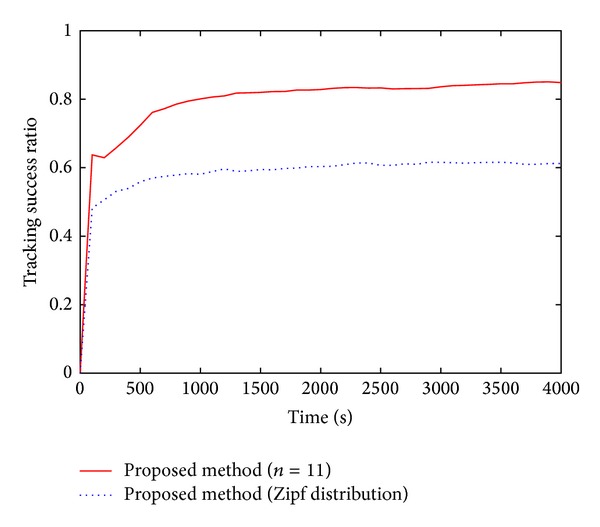
Transitions of tracking success ratio under two distributions of the number of transit microcells.

Figure 4 illustrates the transitions of the tracking success ratio under a fixed number of transit microcells *n* = 11 and that under a Zipf distribution of the number of transit microcells. As shown in Figure 4, the tracking success probability does not almost change after 2000 [s] independently of the methods. This indicates that the system reaches a steady state at 2000 [s].

The tracking success ratio of our proposed method under the Zipf distribution decreases 25–40% compared with that under a fixed number of transit microcells. Since pedestrians with small values of *n* increase in the microcell, the reutilization of velocity information cannot effectively work.

### 4.4. Comparative Evaluations

**Figure 5:**
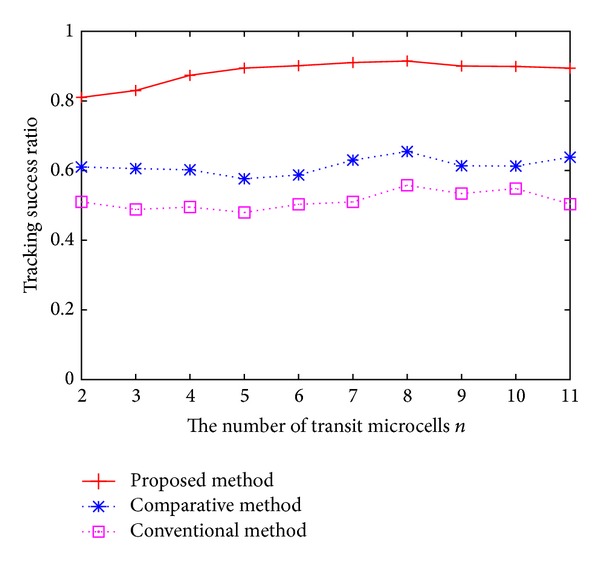
Tracking success ratio against the number of transit microcells.

Finary, we evaluate the effect for obtaining velocity information.  Figure 5 illustrates the relationship between the number of transit microcells *n* and the tracking success ratio when our proposed method, the comparative method, and the conventional method are used. We used a uniform distribution for the number of pedestrians' transit microcells. For the comparative method, *γ* is set to 0.5.

As shown in Figure 5, our proposed method outperforms the conventional method and the comparative method regardless of the number of transit microcells. Our proposed method can improve the tracking success ratio by up to 35% compared to the conventional method by estimating pedestrian trajectories using obtained velocity information in the previous microcell. In addition, our proposed method improves the tracking success ratio by up to 28% compared to the comparative method by determining velocity information based on matching reliability.

## 5. Conclusions and Future Work

In this paper, we proposed a pedestrians tracking method in buildings using a binary sensor network. In our proposed method, sensor nodes are placed at gates, such as doors, stairs, and elevators, to detect pedestrian arrival and departure events. The monitoring area is divided to microcells by gate. Tracking pedestrians in each microcell is conducted by matching the pedestrian gate arrival and gate departure events based on a Bayesian estimation-based method. To improve accuracy of pedestrian tracking, estimated pedestrian velocity and its reliability in a microcell are used for estimating the trajectory of the pedestrian in the succeeding microcell. Through simulation experiments, it was shown that the accuracy of pedestrian tracking using our proposed method is improved by up to 35% compared to a conventional method.

As future work, we plan to evaluate our proposed method in comparison with other pedestrian tracking methods in terms of accuracy, cost, and so forth, using some realistic scenarios. In addition, we also plan to improve our proposed method through implementation and experimental evaluations using off-the-shelf sensor nodes in a real building environment.

## Figures and Tables

**Table 1:** Distance between gates [M].

	*g*_1_	*g*_2_	*g*_3_	*g*_4_	*g*_5_
*g*_1_	0	13.53	5.94	12.21	8.58
*g*_2_	13.53	0	12.87	19.14	15.18
*g*_3_	5.94	12.87	0	7.92	4.29
*g*_4_	12.21	19.14	7.92	0	6.27
*g*_5_	8.58	15.18	4.29	6.27	0

**Table 2:** Transition probability between gates.

(Arrival/departure)	*g*_1_	*g*_2_	*g*_3_	*g*_4_	*g*_5_
*g*_1_	0	0.2	0.4	0.3	0.1
*g*_2_	0.1	0	0.2	0.3	0.4
*g*_3_	0.2	0.1	0	0.1	0.6
*g*_4_	0.1	0.2	0.3	0	0.4
*g*_5_	0.1	0.2	0.5	0.2	0
